# First Report of a Low-Frequency Mosaic Mutation in the Hydroxymethylbilane Synthase Gene Causing Acute Intermittent Porphyria

**DOI:** 10.3390/life13091889

**Published:** 2023-09-10

**Authors:** Adrian Belosevic, Anna-Elisabeth Minder, Morgan Gueuning, Franziska van Breemen, Gian Andri Thun, Maja P. Mattle-Greminger, Stefan Meyer, Alessandra Baumer, Elisabeth I. Minder, Xiaoye Schneider-Yin, Jasmin Barman-Aksözen

**Affiliations:** 1 Institute of Laboratory Medicine, Stadtspital Zürich, Triemli, 8063 Zurich, Switzerland; 2 Division of Endocrinology, Diabetology, Porphyria and Clinical Nutrition, Stadtspital Zürich, Triemli, 8063 Zurich, Switzerland; 3 Swiss Reference Centre for Porphyrias, Stadtspital Zürich, Triemli, 8063 Zurich, Switzerland; 4 Department of Research and Development, Blood Transfusion Service Zurich, Swiss Red Cross, 8952 Schlieren, Switzerland; 5 Department of Molecular Diagnostics and Cytometry, Blood Transfusion Service Zurich, Swiss Red Cross, 8952 Schlieren, Switzerland; 6 Institute of Medical Genetics, University of Zürich, 8952 Schlieren, Switzerland

**Keywords:** acute porphyrias, acute intermittent porphyria, de novo mutation, mosaic mutation, nanopore sequencing

## Abstract

Acute porphyrias are a group of monogenetic inborn errors of heme biosynthesis, characterized by acute and potentially life-threatening neurovisceral attacks upon exposure to certain triggering factors. Biochemical analyses can determine the type of acute porphyria, and subsequent genetic analysis allows for the identification of pathogenic variants in the specific gene, which provides information for family counselling. In 2017, a male Swiss patient was diagnosed with an acute porphyria while suffering from an acute attack. The pattern of porphyrin metabolite excretion in urine, faeces, and plasma was typical for an acute intermittent porphyria (AIP), which is caused by inherited autosomal dominant mutations in the gene for hydroxymethylbilane synthase (HMBS), the third enzyme in the heme biosynthetic pathway. However, the measurement of HMBS enzymatic activity in the erythrocytes was within the normal range and Sanger sequencing of the *HMBS* gene failed to detect any pathogenic variants. To explore the molecular basis of the apparent AIP in this patient, we performed third-generation long-read single-molecule sequencing (nanopore sequencing) on a PCR product spanning the entire *HMBS* gene, including the intronic sequences. We identified a known pathogenic variant, c.77G>A, p.(Arg26His), in exon 3 at an allelic frequency of ~22% in the patient’s blood. The absence of the pathogenic variant in the DNA of the parents and the results of additional confirmatory studies supported the presence of a de novo mosaic mutation. To our knowledge, such a mutation has not been previously described in any acute porphyria. Therefore, de novo mosaic mutations should be considered as potential causes of acute porphyrias when no pathogenic genetic variant can be identified through routine molecular diagnostics.

## 1. Introduction

Acute intermittent porphyria (AIP, OMIM # 176000) is a rare inborn error of heme biosynthesis, characterised by potentially lethal acute neurovisceral attacks [1,2,3]. This inherited autosomal dominant disorder is caused by pathogenic variants in the gene for hydroxymethylbilane synthase (*HMBS*), the third enzyme in the heme biosynthetic pathway. Acute attacks usually present after puberty upon exposure to certain triggering factors that induce hepatic heme biosynthesis, such as drugs metabolised via the cytochrome P450 pathway, exogenous and endogenous hormones, stress, and reduced carbohydrate intake. Biochemically, the hallmark of an acute attack is the increased urinary excretion of heme precursors, including aminolevulinic acid (ALA) and porphobilinogen (PBG), and different porphyrins. Recently published consensus definitions from the working group of the European porphyria network state that an attack of acute porphyria is characterised by having two or more of the clinical manifestations listed in Table 1, persisting for more than 24 h in the absence of other likely explanations. Additionally, there should be a urinary PBG to creatinine ratio of typically more than 10 times the upper limit of normal [3]. According to the currently postulated pathophysiological model, ALA acts as a neurotoxin and causes, amongst other symptoms, severe abdominal pain requiring opioid treatment, nausea, obstipation, muscle weakness, paralysis, electrolyte disturbances (such as hyponatremia), and psychiatric symptoms. However, the exact pathophysiology of an acute attack is not yet completely understood and heme deficiency has been discussed as an alternative or additional factor causing the observed symptoms [4,5,6,7]. Acute attacks can be effectively inhibited by the intravenous administration of heme (hemin, heme arginate), which decreases the expression of aminolevulinate synthase 1 (ALAS1), the first and rate-limiting enzyme of hepatic heme biosynthesis [8]. For a subgroup of patients with recurrent attacks, a treatment option based on RNA interference directed against ALAS1 messenger RNA in the liver has recently been approved [9].

**Figure 1:**
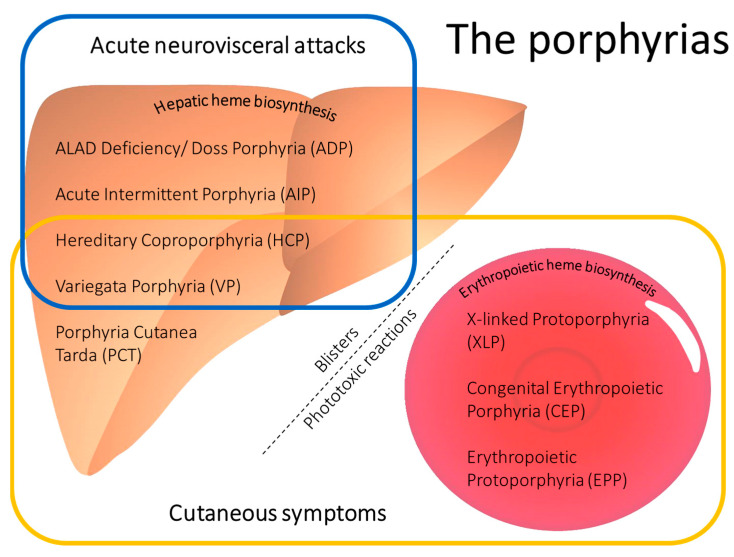
Main clinical characteristics of the porphyrias. The porphyrias are classified as either hepatic or erythropoietic, depending on the main site of porphyrin accumulation. The subgroup of acute porphyrias (within the blue frame) is characterized by acute neurovisceral attacks upon exposure to certain triggering factors that induce hepatic heme biosynthesis. The porphyrias leading to cutaneous symptoms are indicated by the yellow frame. Hepatic porphyrias associated with cutaneous symptoms lead to skin fragility and fluid-filled blisters. Erythropoietic porphyrias are characterised by painful phototoxic reactions after exposure to visible light. In addition, congenital erythropoietic porphyria (CEP) can present with blistering lesions, scars, and mutilations.

An indistinguishable clinical presentation of acute neurovisceral attacks is also caused by heterozygous mutations in the genes for either coproporphyrinogen oxidase (*CPOX*) or protoporphyrinogen oxidase (*PPOX*), leading to hereditary coproporphyria (HCP, # 121300) and variegata porphyria (VP, # 176200), respectively [1,2,3] (Figure 1). In addition, very rarely, homozygous or compound heterozygous missense mutations in the gene for aminolevulinate dehydratase (ALA), the second enzyme in the heme biosynthesis pathway, can cause ALAD deficiency porphyria (ADP, also called Doss porphyria, # 612740). ADP usually manifests during childhood and is characterised by increased ALA (but not PBG). These four inborn errors of heme biosynthesis constitute the group of acute porphyrias (Figure 1, blue frame). For a comprehensive description of the disease characteristics and diagnostic strategies for all forms of porphyria, see the recent review by Neeleman et al. 2020 [10].

The different forms of porphyrias can be differentiated by testing for their characteristic excretion patterns of heme precursors, including ALA and PBG, and porphyrins in urine, blood, and faeces, by measuring enzymatic activities in erythrocytes, and by conducting a plasma fluorescence scan [1,10,11]. These assays are all part of the standard diagnostic protocol used by the Swiss Reference Centre for Porphyrias in Zurich. Information on the clinical symptoms at the time the samples were taken is necessary. For example ALA, PBG, and urinary porphyrin excretions can quickly normalize after an acute attack [12]. Conversely, in patients with a high excreter phenotype, increased urinary excretions of ALA, PBG, and porphyrins can be found even several years after the clinical symptoms of the acute attack have been resolved. The plasma fluorescence scan is the screening test used for all porphyrias associated with cutaneous symptoms (see Figure 1, yellow frame). The maximum emission wavelengths (peaks) already provide information to identify the underlying form of porphyria. Interestingly, in AIP, which is usually not associated with cutaneous symptoms, a positive plasma fluorescence scan signal can be found in patients during an acute attack and even in a subgroup of patients in remission [13,14]. Although the management of the acute attack does not depend on the underlying form of acute porphyria, determination of the type of acute porphyria enables the mutational analysis of the corresponding gene for family counselling. Family members who inherit the pathogenic variant are susceptible to acute attacks when exposed to triggering factors and have an increased risk of developing hepatocellular carcinoma (HCC), one of the long-term complications associated with acute porphyrias. Therefore, the Swiss Reference Centre for Porphyrias offers counselling and genetic analysis to the family members of index patients with acute porphyrias. This has been shown to reduce the risk of developing acute attacks and the likelihood of a severe disease course and enables life-saving screening for early-stage HCC [15,16,17,18,19].

In this study, we describe an adult male patient with acute porphyria who was diagnosed in 2017 during an acute attack, based on increased urinary excretion of ALA, PBG, and porphyrins. Further biochemical analyses, performed by the Swiss Reference Centre for Porphyrias, were consistent with a diagnosis of AIP (see the results for a detailed description of AIP). However, there was no reduction in HMBS enzymatic activity as measured in blood erythrocytes 3 months after the acute attack (see Section 3). In general, HMBS activity is a reliable marker for the presence of pathogenic variants in the HMBS gene. Together with the differentiation of faecal porphyrins, it can be used to identify patients with AIP during the latent phase, when urinary porphyrin, ALA, and PBG excretion might have already normalized. False normal erythrocyte HMBS activity can be found in patients undergoing frequent blood draws (for example, during an inpatient stay in hospital). This process stimulates erythropoiesis and leads to a higher proportion of young erythrocytes in the blood, which have higher HMBS activity [20]. Alternatively, mutations in exon 1, which is expressed in the liver but not in erythrocytes, could lead to normal HMBS enzymatic activity in blood samples but reduced enzymatic activity in the liver [21,22]. However, in our patient, Sanger sequencing of the *HMBS* gene (including exon 1) did not lead to the identification of a pathogenic variant. In addition, sequencing of the genes for *PPOX* and *CPOX* did not result in a molecular diagnosis. As a result of a request for genetic counselling by the family, we re-examined the patient’s DNA in 2022 using a third-generation long-read single-molecule sequencing approach (nanopore sequencing, Oxford Nanopore Technologies (ONT)). This allowed us to analyse the entire *HMBS* gene, including its intronic and regulatory regions.

## 2. Materials and Methods

### 2.1. Informed Consent

Written informed consent was obtained from the patient and his parents prior to conducting the genetic analyses, in accordance with Swiss legislation. The index patient also signed a written informed consent form for participation in the porphyria biobank project, which was approved by the Cantonal Ethics Committee in Zurich (BASEC 2018-00758), and further provided a separate written informed consent form for the publication of this case report.

### 2.2. Biochemical Analyses

The quantification of ALA and PBG was performed using the ClinEasy^®^ kit (Recipe Chemicals + Instruments GmbH, Munich, Germany) from native urine samples. The differentiation and quantification of urinary and faecal porphyrins, the plasma fluorescence scan, and HMBS enzymatic activity assays were performed according to previously published methods [11]. The Swiss Reference Centre for Porphyrias is a recognized specialist centre of the Nationale Koordination Seltene Krankheiten (kosek) and the European Porphyria Network, and all the laboratory methods listed under Section 2.2 and Section 2.3 (Sanger sequencing) are accredited by the Swiss Accreditation Service (SAS), according to the ISO norm 15,189 for medical laboratories.

### 2.3. Sanger Sequencing

Whole blood samples were collected in EDTA tubes, and DNA was extracted and amplified with respect to the exonic sequences of the *HMBS*, *PPOX* and *CPOX* genes. Subsequently, Sanger sequencing was performed, as previously described [23]. The generated sequences were visualized using Sequencing Analysis Software v7.0 (Applied Biosystems, Waltham, MA, USA). Using the Variant Reporter Software v3.0 (Applied Biosystems) program, the generated sequences were compared to reference sequences NC-000011.10, NC-000001.11, and NC-000003.12 from the National Center for Biotechnology Information (NCBI). In addition, all the sequences were analysed visually.

### 2.4. Long-Range PCR

To prevent primer mismatches and potential allelic dropout during PCR amplification, an investigation was performed using the NCBI database to examine the presence of single nucleotide polymorphisms (SNPs) in the intronic and flanking regions of the *HMBS* gene on chromosome 11, position 119,066,840–119,136,258 (NCBI Reference Sequence: NC-000011.10) in dbSNP. Prior to long-range PCR (LR-PCR) primer design, the presence of these SNPs in the DNA sample of the patient was investigated using Sanger sequencing. The primers were designed using the Primer-BLAST tool from the NCBI and were ordered from Microsynth AG (Balgach, Switzerland). The LR-PCR amplicon was generated with the forward primer situated in the promoter region of the *HMBS* gene, and the reverse primer in the UTR of exon 15, generating a PCR product of 8′716 bp spanning the entire region of interest. The primer sequences are provided in Table 2.

The LR-PCR was performed using PrimeSTAR GXL DNA polymerase TaKaRa Bio with 1 M Betaine enhancer (VWR) on a 60 well Veriti™ Thermal Cycler (Applied Biosystems) platform, as previously described [24]. The LR-PCR products were examined on an agarose gel (1%) in Tris-acetate-EDTA (TAE) buffer with a MassRuler High Range DNA Ladder (Thermo Fisher Scientific, Allschwil, Switzerland) and were visualised using an Azure c200 Gel Imagine System (Azure Biosystems, Dublin, CA, USA). The LR-PCR products were purified using 1X Agencourt AMPure XP magnetic beads (Beckman Coulter, Brea, CA, USA). The purified LR-PCR products were quantified using a dsDNA broad range assay kit on a Qubit fluorometer 4.0 (Invitrogen, Waltham, MA, USA).

### 2.5. Nanopore Sequencing

The sequencing library preparation of the LR-PCR product was conducted in four steps, following the protocol from Oxford Nanopore Technologies (ONT: “Amplicon barcoding with Native Barcoding Expansion 96”; version: NBA_9102_v109_revJ_09Jul2020). A barcoding approach was used to enable parallel sequencing of the index patient’s sample, along with the samples of three other patients. The PCR products were end-repaired using the NEBNext Ultra II End repair/dA-tailing Module (New England BioLabs, Ipswich, MA, USA) to get rid of DNA overhangs produced during the PCR. In the next step, barcodes that were specific for each patient were attached to both ends of the respective PCR product, and sequencing adapters were ligated to the barcodes. The final library was loaded into a Flongle flow cell (R9.4.1, ONT), which was then placed in a MinION sequencer (ONT). Sequencing was stopped after 24 h. Basecalling was conducted using the Guppy (v.5.1.15) algorithm from the MinKNOW software (v.21.11.9, ONT). Simultaneously, the reads were grouped based on the barcodes (demultiplexing), generating FASTQ format files for each patient. This was followed by filtering FASTQ reads with a length of 7500 bp–10,000 bp, as the sequence of interest was 8716 bp long. Using PoreChop (v0.2.3), the remaining barcode and adapter sequences were trimmed. Minimap2 (v.2.17) was used to map the filtered and trimmed reads to the reference sequence for *HMBS* (NG_008093). By using samtools (v.1.15), the aligned reads were sorted, indexed, and converted into BAM format files, which could be visualised in Integrative Genome Viewer (IGV v.2.10). Finally, these files were used for variant calling, where variants between the reference sequence and the sequence reads were output in Variant Calling Format (VCF). Two separate tools were used to generate VCF format files, Medaka (v.1.2.3) and Clair (v.0.1). IGV was used to inspect the allele frequencies of variant calls. Additionally, the aligned sequences were further visually examined in IGV to detect the variants that were potentially missed by the two variant calling tools. Purification and quantification of the LR-PCR products, preparation of the library, and the sequencing and analysis of the LR-PCR products were conducted at the Blood Transfusion Service Zurich in Schlieren (Switzerland).

### 2.6. Sequence-Specific Primer PCR

Sequence-specific primer PCR (SSP-PCR) was used to verify the variant with low allele frequency identified in the patient’s DNA using nanopore sequencing. The last nucleotide at the 3′ end of the reverse primer was designed to match only the alternative nucleotide and not the reference nucleotide. Additionally, a second mismatch was introduced two nucleotides prior to the last nucleotide at the 3′ end, which was expected to lead to higher specificity of the reverse primer for the DNA sequence containing the alternative nucleotide. The primer sequences are provided in Table 3.

### 2.7. Analysis of Short Tandem Repeats

To distinguish between chimerism and mosaicism, a quantitative fluorescence polymerase chain reaction (QF-PCR) was performed to analyse 26 short tandem repeats (STRs) located on chromosomes 13, 18, 21, X, and Y. The results were analysed using GeneMapper Software 5. The QF-PCR was conducted and analysed at the Institute of Medical Genetics (University of Zurich) in Schlieren (Switzerland).

## 3. Results and Discussion

Here and for the first time, we report a mosaic mutation causing AIP. The adult male patient developed a severe acute porphyria attack after a viral infection and treatment with potentially porphyrinogenic medications (mefenamic acid and metamizole). The acute attack was associated with abdominal pain, nausea, constipation, restlessness, disorientation, hypertensive derailment (crisis), and hyponatremia and required intensive care treatment. After successful treatment of the acute attack with heme arginate, he was referred to the outpatient clinic of the Swiss Reference Centre for Porphyrias for counselling regarding the diagnosis, triggering factors, possible long-term complications of acute porphyria, and family testing. According to the patient, he had previously never experienced any symptoms of an acute porphyria attack and also had no family history of the condition.

The results of the biochemical analyses three months after the initial attack, i.e., the porphyrin excretions in the urine and faeces and the positive plasma fluorescence scan at 619 nm, indicated an AIP with a high excretion phenotype pattern. However, the HMBS enzymatic activity, as measured in the erythrocytes, was within the normal range (Table 4).

Sanger sequencing of the HMBS gene, including exon 1, and subsequently of PPOX and CPOX, did not lead to the identification of a pathogenic variant. However, the Sanger sequencing approach used in the laboratory of the Swiss Reference Centre for Porphyrias only covers the exonic and exon–intron boundary sequences and does not include the full promotor regions. Therefore, deep intronic variants, large deletions and insertions, and mutations located in the housekeeping promotor region of the HMBS gene might have remained undetected.

**Figure 2:**
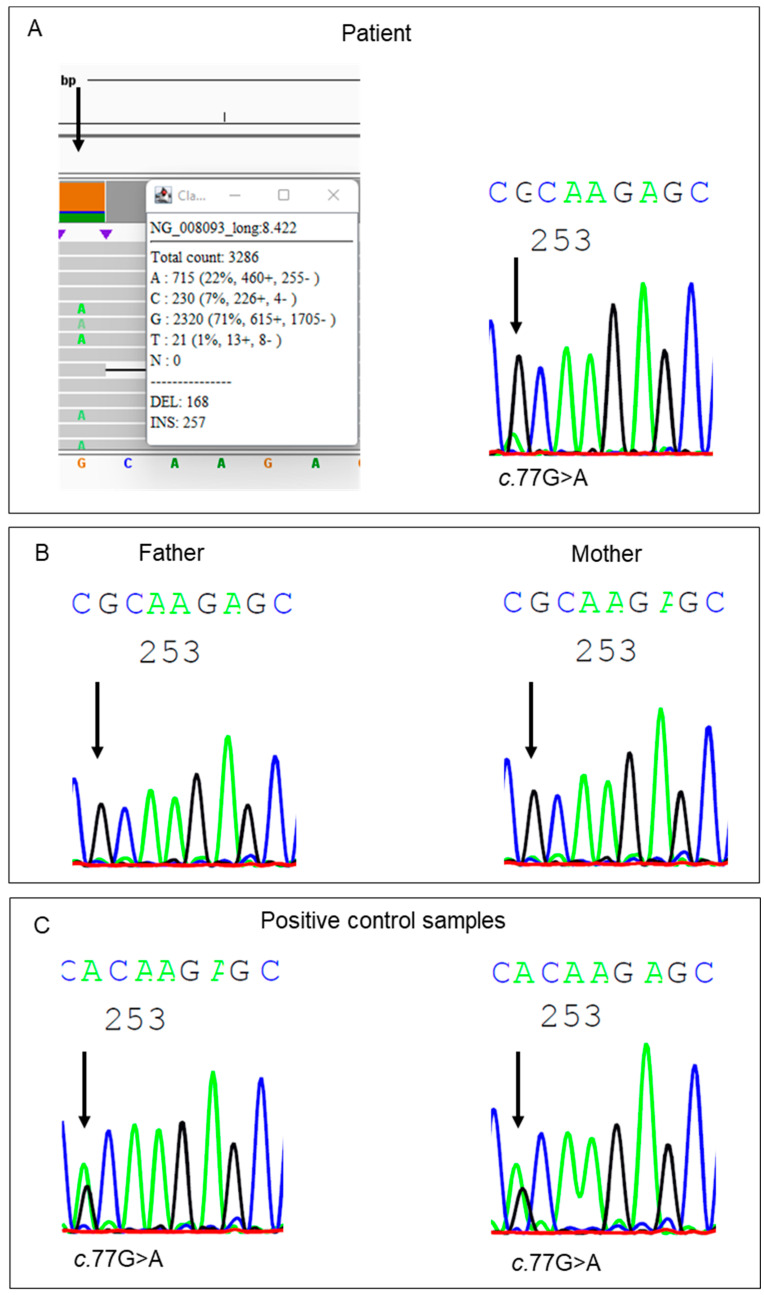
Results of the mutational analyses of the *HMBS* gene generated by nanopore or Sanger sequencing, focusing on the c.77G region in exon 3. (**A**) The left column shows the nanopore sequencing result with a 22% allele frequency of the pathogenic c.77G>A variant. A low intensity signal for the same variant is detectable in the results obtained using Sanger sequencing. (**B**) Sanger sequencing of the patient’s parents shows no signal for the c.77G>A variant. (**C**) A strong intensity signal for the c.77G>A variant was detected in two positive controls using Sanger sequencing.

Because genetic counselling was requested by the family, measurements of HMBS enzymatic activity were also performed on the blood samples obtained from the parents and were repeated in the index patient in 2021. This time, the HMBS activity measured in the blood sample of the patient was borderline reduced (88% LLN; Table 4B). The HMBS activities in the blood samples of both parents were within the normal range. Meanwhile, new long-read single-molecule sequencing approaches had become available, allowing for the analysis of the full intronic and promotor regions of a single gene, including the ability to detect insertions and large deletions [24]. In a new attempt to identify the underlying pathogenic variant, we performed LR-PCR on the *HMBS* gene using freshly isolated DNA from the patient. Nanopore sequencing of the amplicons revealed a variant in exon 3 in ~22% of the sequenced reads. The identified single nucleotide substitution, c.77G>A, p.(Arg26His), is a known pathogenic variant that has been previously described in the literature [26]. Re-examination of the previously generated Sanger sequencing results showed a small but discernible signal for the identified substitution (Figure 2A). Expecting a 50% allele frequency for this usually hereditary disorder, the small signal detected on the electropherogram had initially been interpreted as noise. The variant was also not detected by the software-assisted sequencing analysis, which is part of the standard analysis procedure in the laboratory. The presence of the variant in the DNA sample of the patient and two positive control samples was confirmed using PCR with sequence-specific primer pairs (Figure 2C). Analysis of the DNA of the parents with Sanger sequencing (Figure 2B) and PCR with sequence-specific primer pairs showed the absence of the variant in their blood samples.

Variants detected at low allele frequencies can be caused by chimerism, in which cells originating from different zygotes merge during an early embryonic phase, or are due to stem cell transplantation [27]. Alternatively, such low variant allele frequencies can be caused by somatic de novo mutations, leading to a mosaic distribution of the variant [28,29]. To distinguish between potential mosaicism and chimerism, an analysis of 26 short random repeats was performed. This analysis showed only two different microsatellite lengths for all investigated loci. Therefore, we excluded chimerism as a possible explanation. The DNA used for our analysis was extracted from white blood cells and originates from cells in the bone marrow, which develop from the mesoderm, whereas the liver originates from the endoderm. The low allele frequency of the pathogenic variant, as determined using the blood samples, is consistent with the absence of borderline reduced HMBS enzymatic activity found in the erythrocytes 3 months and 3.5 years after the acute attack. However, it does not give an indication about the residual enzymatic activity in the liver. Given the clinically clear presentation of an acute attack, we speculate that the enzymatic activity of HMBS in the liver significantly decreased. However, as investigating liver samples is invasive and, hence, was understandably not feasible, the analysis was limited to the DNA samples derived from other organs not involved in the pathophysiology of AIP. Since a classical acute porphyria attack led to the diagnosis of an acute porphyria, counselling of the patient regarding disease management does not differ from that of patients carrying germline mutations. By avoiding triggering factors, he remained free of any further acute attacks until now. Transmission of the condition to the next generation cannot be excluded, as the pathogenic variant might be present in the germline cells of the index patient [30]. Therefore, the children of the patient should be tested for the presence of the identified pathogenic variant. To our knowledge, the presented case is the first description of mosaicism in AIP.

## 4. Conclusions

In the absence of pathogenic variants in the HMBS gene in DNA derived from blood samples, de novo mosaic mutations in the liver should be considered as a potential cause of AIP. In case the de novo mosaic mutation also affects the germline cells of the index patient, transmission to the next generation is possible and children should be tested for the presence of the pathogenetic variant.

## Figures and Tables

**Table 1:** Clinical manifestations of an acute porphyria attack (modified from [3] and own observations).

An Acute Porphyria Attack Is Characterised by:	
Two or More of the Following Clinical Manifestations, Typically Persisting > 24 h:	AND Increased Urinary Porphobilinogen (PBG):
- Severe abdominal pain in the absence of any alterations in routine diagnostic markers (laboratory tests and imaging). Pain in the back, chest, and extremities can also be present	Urinary PBG * to creatinine ratio of typically more than 10 times the upper limit of normal
- Nausea, vomiting, and/or constipation	
- Hypertension and tachycardia	
- Hyponatraemia	
- Peripheral neuropathy associated with symptoms such as muscle weakness, paralysis, reduced tendon reflexes, and respiratory arrest	
- Urinary retention or incontinence. Urine can be concentrated and may show reddish/brownish discoloration when exposed to light and oxygen	
- Symptoms affecting the central nervous system, such as irritability, seizures (typically associated with hyponatremia), confusion, reduced consciousness, psychosis, or posterior reversible encephalopathy syndrome on an MRI scan	

* In the very rare aminolevulinate dehydratase deficiency porphyria (ADP), urinary porphyrins and aminolevulinic acid are increased (not PBG).

**Table 2:** Sequences for the long-range PCR primers.

Forward primer (5′-3′)	GCAAAGGAAGCGCCATAGAAG
Reverse primer (3′-5′)	AGGCAAGGCAGTCATCAAGG

**Table 3:** Sequence-specific primer for verification of the variant with low allele frequency. The alternative nucleotide and the matching nucleotide in the reverse primer sequence are highlighted in red. The additional mismatch is highlighted in blue.

Sequence with reference nucleotide (5′-3′)	ACCC**G**CAAGAGCCAGGTGGGTGCAGGAG
Sequence with alternative nucleotide (5′-3′)	ACCC**A**CAAGAGCCAGGTGGGTGCAGGAG
Reverse primer with mismatch (3′-5′)	**T**G**A**TCTCGGTCCACCCACGT

**Table 4:** Laboratory parameters. (**A**) Biochemical parameters suggestive of an acute intermittent porphyria. (**B**) HMBS enzymatic activity.

(**A**)					
	**Aminolevulinic Acid (urine)**	**Porphobilinogen (urine)**	**Plasma Fluorescence Scan**	**Faecal Coproporphyrin III ^(1)^**	**Faecal Protoporphyrin ^(2)^**
Reference range	<2.5 [μmol/mmol creatinine]	<1.25 [μmol/mmol creatinine]	Pos/neg [nm]	<12.0 [mmol/g dry weight]	<80 [mmol/g dry weight]
During the acute attack	76.4	35.2			
After the acute attack	15.5 ^(a)^ 12.5 ^(b)^	22.4 ^(a)^ 25.0 ^(b)^	619 ^(a) (c)^	3.57 ^(c)^	16.9 ^(c)^
(**B**)					
	**Reference Range**	**Healthy Controls (n = 20), Mean (SD) ^(3)^**	**AIP Patients (n = 16), Mean (SD) ^(3)^**	**Patient 1st Measurement**	**Patient 2nd Measurement**
HMBS enzymatic activity	66–126 [pmol/mgHb/h]	99.6 (±27.8)	44.9 (±9.0)	85 ^(c)^	58 (88% LLN) ^(a)^

^(1)^ An isolated increased faecal coproporphyrin III would indicate hereditary coproporphyria [1]; ^(2)^ increased faecal protoporphyrin (together with coproporphyrin III) would indicate porphyria variegata [1]; ^(a)^ 3.5 years after the acute attack; ^(b)^ 5.5 years after the acute attack; and ^(c)^ 3 months after the acute attack. Elevated aminolevulinic acid and porphobilinogen 3.5 and 5.5 years after the acute attack indicates a high excreter phenotype. ^(3)^ HMBS enzymatic activities in healthy controls and patients with confirmed AIP from the Swiss Reference Centre for Porphyrias, as previously published [25]. AIP: acute intermittent porphyria; LLN: lower limit of normal.

## Data Availability

Data that does not put the patient at risk for deanonymization can be obtained upon reasonable request from the corresponding author.
